# Prevalence and predictors of ICD‐11 posttraumatic stress disorder and complex PTSD in young people

**DOI:** 10.1111/acps.13442

**Published:** 2022-05-11

**Authors:** Enya Redican, Philip Hyland, Marylene Cloitre, Orla McBride, Thanos Karatzias, Jamie Murphy, Lisa Bunting, Mark Shevlin

**Affiliations:** ^1^ School of Psychology Ulster University Coleraine UK; ^2^ Department of Psychology Maynooth University Maynooth Ireland; ^3^ National Center for PTSD Verterans Affairs Palo Alto Health Care System Palo Alto California USA; ^4^ School of Health and Social Care Edinburgh Napier University Edinburgh UK; ^5^ School of Social Sciences, Education and Social Work Queen's University Belfast UK

**Keywords:** child and adolescent International Trauma Questionnaire, Complex PTSD, factor mixture modelling, ICD‐11, Posttraumatic stress disorder

## Abstract

**Objective:**

The prevalence, construct validity, risk factors and psychopathological correlates associated with ICD‐11 posttraumatic stress disorder (PTSD) and complex PTSD (CPTSD) as measured by the International Trauma Questionnaire for Children and Adolescents (ITQ‐CA) were assessed in a sample of young people from Northern Ireland.

**Method:**

Participants were trauma‐exposed 11–19‐year‐olds (*N* = 507) who participated in the Northern Ireland Youth Wellbeing Prevalence Survey (YWS‐NI, 2020). Factor mixture modelling (FMM) was used to test the latent structure of the ITQ‐CA. Risk‐factors and psychopathological correlates associated with latent class membership, and ICD‐11diagnostic status, were also investigated.

**Results:**

More participants met the ITQ‐CA criteria for CPTSD (3.4%, *n* = 44) than PTSD (1.5%, *n* = 19). A second‐order FMM comprising a ‘partial‐PTSD class’, a ‘CPTSD class’, a ‘DSO class’ and a ‘low symptom endorsement class’ was the best‐fitting model. Younger age and cumulative trauma were risk factors for all trauma classes. Female gender and two or more violent traumas were significant predictors of the ‘PTSD’ and ‘CPTSD’ classes, while single sexual trauma was a significant predictor of the ‘DSO’ and ‘CPTSD’ classes. Two or more sexual traumas was a unique predictor of ‘CPTSD class’, while two or more vicarious traumas was a unique predictor of ‘DSO class’. The ‘CPTSD’ class displayed the most notable comorbidity.

**Conclusions:**

Findings indicate that CPTSD may be more prevalent than PTSD in children and young people. Support for the ICD‐11 conceptualisation of CPTSD as representing a unique diagnostic construct was supported using FMM, with findings indicating trauma symptom class‐specific risk profiles.

**Significant outcomes**

- This is the first study to use factor mixture modelling to investigate the validity of ICD‐11 PTSD and CPTSD as measured by the ITQ‐CA.
- Findings align with existing studies supporting the psychometric properties of the ITQ‐CA scores.
- Results indicate that both trauma type and quantity of traumatic exposure are important predictors of PTSD and CPTSD.

**Limitations**

- The use of a general‐population sample limits generalisability to treatment‐seeking samples.
- Anxiety and depression were the only psychological outcomes assessed, however, there are other disorders also associated with PTSD and CPTSD.
- Cross‐sectional nature of study limits inferences regarding causality.

## 1 INTRODUCTION

Posttraumatic stress disorder (PTSD) and complex PTSD (CPTSD) are included in the 11th version of the International Classification of Diseases (ICD‐11) as trauma‐related disorders.^1^ The PTSD diagnosis includes the three symptom clusters of (1) re‐experiencing of the trauma in the here and now (Re), (2) avoidance of traumatic reminders (Av), and (3) sense of current threat (Th). The CPTSD diagnosis includes the core PTSD symptoms and three symptom clusters of (1) affective dysregulation (AD), (2) negative self‐concept (NSC) and (3) disturbed relationships (DR), which collectively represent ‘Disturbances in Self‐Organisation’ (DSO).^2^ The International Trauma Questionnaire (ITQ)^3^ and the ITQ for children and adolescents (ITQ‐CA)^4^ have been designed to reflect the diagnostic profiles of PTSD and CPTSD as described in ICD‐11. Although the construct validity of the ITQ is well‐established in adult populations, where confirmatory factor analytic (CFA) studies have supported the factorial validity of the ITQ and mixture modelling studies have supported the conceptual distinctiveness of PTSD and CPTSD (for review, see Reference 5), research on young people using the ITQ‐CA is less extensive.

The construct validity of the ITQ‐CA was first investigated in a sample of Lithuanian adolescents aged 12–16 years^6^ where a correlated six‐factor model—with factors representing the six symptom clusters described above—was the best‐fitting model while another study conducted on Austrian foster children aged 10–18 years found a two‐factor second‐order model—where the second‐order factors represented ‘PTSD’ and ‘DSO’—as superior.^7^ Notably, the PTSD and DSO factors were highly correlated in the latter study, while the correlated six‐factor model and a one‐factor higher‐order CPTSD model also provided good fit. The adequacy of several models has led to suggestions that the developmental epoch of the sample may influence the latent structure of the ITQ‐CA given that adolescence is a highly sensitive period which encompasses many biopsychosocial changes.^6^, ^7^ Alternatively, the nature of the investigated population (i.e., clinical or general‐population) may influence the latent structure of the ITQ‐CA,^6^, ^7^ similar to the adult literature where the correlated six‐factor model has been most supported in general‐population samples and the second‐order model in highly‐traumatised treatment‐seeking samples.^5^ Given that identifying the correct latent structure bears important influences for the resulting diagnostic algorithm,^8^ establishing the latent structure of the ITQ‐CA is paramount. The high correlation observed between PTSD and DSO has also raised some concerns regarding distinguishability of these symptom clusters in young people,^7^ a matter which requires further attention. The conceptual distinctiveness of PTSD and CPTSD using the ITQ‐CA has been examined and supported in only one study.^6^ Recently, factor mixture modelling (FMM), a hybrid model which allows the latent structure of a psychological construct to be both dimensional (factor analysis) and categorical (mixture modelling),^9^ was used to evidence the conceptual distinctiveness of PTSD and CPTSD in US adults.^10^ However, no such study has been conducted using the ITQ‐CA in young people which may prove instrumental in determining the validity of ICD‐11 PTSD and CPTSD in young people.

Establishing the prevalence and correlates of ICD‐11 PTSD and CPTSD across different countries and cultures is pivotal to determine cross‐cultural applicability.^11^ Northern Ireland (NI) is a particularly suitable candidate for such research given the historical context of ‘the Troubles’, a period of political and civil conflict in NI which spanned across 30 years.^12^, ^13^ Largely attributed to ‘the Troubles’, the prevalence of PTSD in the adult NI population is among the highest internationally^14^ and emerging evidence indicates an intergenerational transmission of trauma stemming from this period.^15^, ^16^ The only general‐population study to investigate ICD‐11 PTSD and CPTSD prevalence in young people was conducted in Lithuania where the PTSD prevalence was 10.4% and CPTSD prevalence was 11.6%.^17^ These estimates are relatively higher than other general‐population studies among adults^18^, ^19^, ^20^ and thus, establishing prevalence rates across different populations of young people is necessary. There is a dearth of research investigating factors which increase a young person's susceptibility to CPTSD however, interpersonal trauma,^6^, ^7^, ^17^, ^21^ cumulative trauma,^7^, ^19^, ^22^ female gender^7^, ^23^ and social factors^17^ have been indicated as particularly salient risk factors. Different patterns of comorbidity have also been observed in young people compared with adult populations,^21^, ^24^ however such research is limited.

### 1.1 Aims of the study

The primary objectives of the current study were to determine the prevalence, construct validity, risk‐factors and psychopathological correlates associated with ICD‐11 PTSD and CPTSD using data from the first ever nationally representative epidemiological survey of mental health for young people living in NI.

## 2 METHODS

### 2.1 Study design

Participants for the current study included a nationally representative sample of 11–19‐year‐olds (*N* = 1293) who participated in the NI Youth Wellbeing Prevalence Survey (YWS‐NI, 2020)^25^ ‐ the first large‐scale study to investigate PTSD and CPTSD prevalence in young people living in NI. The sampling frame was based on the Pointer database, a register comprising of the postcodes of all households in NI. Fieldwork for the YWS‐NI took place between June 2019 and 20th of March 2020. A total of 21,730 addresses were randomly selected, of which 79% were found to be ineligible because of there being no young person residing at the address (83%), the resident status of addresses being unconfirmed (9%) and the addresses being vacant or unable to be found (7%). Of the 4621 eligible households, 67% participated, resulting in a total of 3074 parent or young person surveys being completed for the mental health component of the survey. Only participants aged 11–19 years (*n* = 1299) were asked to complete items pertaining to trauma and stress‐related disorders in the NI‐YWS‐NI, with six of those participants excluded in the current study due to missing data. Full details of the survey methods are available to view at the Open Science Framework.^26^

### 2.2 Participants

Rates of trauma exposure and prevalence estimates of ICD‐11 PTSD and CPTSD and gender differences were calculated for the survey sample (*n* = 1293), while only those who endorsed at least one traumatic stressor were included as the analytic sample (*n* = 509). Of those 509 participants, two were excluded because of excessive missing data on the ITQ‐CA items, leaving a final total of 507. The mean age of the final sample was 15.29 years (*SD* = 2.51, Range = 11–19 years, Median = 15.00). There were more males (53.5%, *n* = 271) than females (46.5%, *n* = 236). More than a third of participants were members of households in receipt of social welfare (37.7%; *n* = 191) and lived with one biological parents (37.4%; *n* = 189); 11.6% (*n* = 58) had special educational needs and, a small proportion (4.3%; *n* = 22) reported experiences of out‐of‐home care. Additionally, 22.3% (*n* = 113) had a parent experiencing clinically significant mental health problems, and 10.7% (*n* = 54) had a parent who reported high levels of adverse childhood experiences (i.e., ≥4 ACEs).

### 2.3 Measures

#### 2.3.1 ICD‐11 PTSD and CPTSD

The ITQ‐CA^4^ is a self‐report measure of ICD‐11 PTSD and CPTSD for young people. Six items assess the PTSD symptom (Re, Av, Th) clusters and six items assess the DSO symptom clusters (AD, NSC, DR). The ITQ‐CA is adapted from the ITQ for adults guided by feedback from three child trauma psychologists with expertise in child measure development. Symptom descriptions were adjusted to be developmentally sensitive to the middle school and adolescent years and the items and instructions revised for a fourth grade reading level (e.g., original ITQ item ‘Being “super‐alert,” watchful, or on guard?’ is ‘Being overly careful (checking to see who is around me)’ in the ITQ‐CA). The young person is asked to indicate their index trauma, and considering that event, the extent to which they have been bothered by each of the PTSD and DSO symptoms in the preceding month using a five‐point Likert scale ranging from ‘never’ (0) to ‘almost always’ (4). The presence of a symptom is indicated with a Likert score ≥2 (i.e., ‘sometimes’). An additional five items rated using yes/no responses assess the severity of functional impairment associated with PTSD and DSO symptomology across various domains of relevance within a young person's ecology (i.e., friendships, family relationships, schoolwork, hobbies and general happiness). Endorsement of one symptom from each PTSD symptom cluster and a functional impairment item is required for PTSD diagnosis. Endorsement of one symptom from each PTSD and DSO symptom cluster as well as a functional impairment item related to PTSD and/or DSO symptomology is required for CPTSD diagnosis. The validity and reliability of the ITQ‐CA has been supported in prior studies.^6^, ^7^ The ITQ‐CA is available freely online.^27^

#### 2.3.2 Trauma exposure

The traumatic events checklist, a 14‐item checklist which forms part of the Child and Adolescent Trauma Screen (CATS),^28^ is a self‐report measure that was used to assess trauma exposure. Items are scored dichotomously as yes (1) or no (0) responses. A full list of the items included in the CATS along with endorsement rates for the survey sample are provided in Data S1. Because of the low endorsement of many traumas and to preserve power, several aggregate trauma categories were created including sexual trauma, exposure to direct harm or violence and vicarious violence exposure. Three traumas were included individually as they were sufficiently distinct from all others. Dummy‐coded variables were created to reflect different levels of exposure within each category (i.e., 0, 1 or ≥2 exposures). The reference class was zero exposures.

#### 2.3.3 Mental health outcomes

The Revised Child Anxiety and Depression Scales (RCADS)^29^ is a 47‐item self‐report measure comprised of six subscales which assesses common mood and anxiety disorders. Six sub‐scales assess separation anxiety disorder (SAD), social phobia (SP), generalised anxiety disorder (GAD), obsessive–compulsive disorder (OCD) and panic disorder (PD), and major depressive disorder (MDD), as defined by DSM‐IV. Items are answered on a four‐point Likert scale ranging from ‘never’ (0) to ‘always’ (3). For the current study, raw scores from each individual sub‐scale were utilised. Cronbach's alpha for each of the sub‐scales were excellent: GAD (α = 0.88), MDD (α = 0.90), PD (α = 0.90), OCD (α = 0.81), SAD (α = 0.76), and SP (α = 0.90).

#### 2.3.4 Predictor variables

Predictor variables included gender (male = 0, female = 1), age (measured in years), experiences of out‐of‐home care including spending time in a children's home, with foster parents, kinship carers, in a secure residential facility, a juvenile justice unit or other (no = 0, 1 = yes), special educational needs (SEN; no = 0, yes = 1), household composition (not living with both biological parents = 0, living with both biological parents = 1), family in receipt of income disability benefits (not in receipt = 0, in receipt of income or disability benefits = 1), and area level deprivation deciles (1–10, with lower scores indicating higher levels of deprivation). The 12‐item General Health Questionnaire (GHQ‐12)^30^ which enquires about the recent presence of symptoms indicative of psychological distress and poor general functioning was completed by a parent and was used to assess parental mental health. Items are responded to on a four‐category scale (e.g., ‘Better than usual’ to ‘Much less than usual’) and was scored using the standard method (0–0–1–1) producing possible scores ranging from 0 to 12 with a score of ≥3 indicative of probable mental health problems. The reliability of the GHQ‐12 in the current study was excellent (α = 0.91). Parent self‐reported ACEs were assessed using the 10‐item Adverse Childhood Experiences questionnaire (ACE).^31^ Items are scored dichotomously, with participants responding either yes (1) or no (0). Based on prior research indicating four or more ACEs to be the threshold for elevated risk of maladaptive outcomes,^31^ parents whose ACE scores ≤3 were allocated to a ‘low ACE score’ (0) group and parents with ACE scores ≥4 were allocated to ‘high ACE score’ (1) group. Parental ACEs were dichotomized in order to isolate the effect of having a parent with a high ACE score on young person outcomes.

### 2.4 Statistical analysis

Using the same approach taken in a prior study,^10^ FMM was conducted in three sequential steps. The first step involved testing four alternative CFA models to determine the latent structure of the ITQ‐CA. Model 1 was a one‐factor model where all 12 PTSD and DSO symptoms loaded onto a first‐order ‘CPTSD’ factor; Model 2 was a correlated six‐factor model where all pairs of PTSD and DSO symptoms loaded onto their respective first‐order factors (Re, Av, Th, AD, NSC, DR), and these factors were correlated; Model 3 was a two‐factor second‐order model where the first‐order Av, Re and Th factors loaded onto the second‐order ‘PTSD’ factor, and the first‐order AD, NSC and DR factors loaded onto the second‐order ‘DSO’ factor and the second‐order factors were correlated; and Model 4 was a one‐factor second‐order model where the six first‐order factors (Av, Re, Th, AD, NSC, DR) all loaded onto the second‐order ‘CPTSD’ factor. Fit indices used to assess goodness of fit included the chi‐square statistic, comparative fit index (CFI),^32^ Tucker‐Lewis's index (TLI),^33^ root mean square of approximation (RMSEA)^34^ and Standardised Root Mean Square Residual (SRMR).^35^ Model fit was assessed using standard criteria,^36^ a non‐significant $x^{2}$ value (*p* ≥ 0.05), CFI and TLI values ≥0.90 and ≥0.95 considered as good and excellent model fit, respectively, RMSEA values <0.05 and SRMR values ≤0.80, all indicated good fit. Lower Bayesian Information Criterion (BIC), sample size adjusted BIC (ssaBIC)^37^ and Akaike Information Criterion (AIC)^38^ values indicated better model fit. The model with the lowest BIC was considered to be the best model with differences greater than 10 being considered strong evidence for the selection of the lower BIC model.^39^ Based on the best‐fitting model, composite reliability (CR) estimates were calculated for the PTSD and DSO sub‐scales. CR provides a more accurate estimate of internal consistency than Cronbach's alpha as it does not rely on the strict assumption of tau‐equivalence of item indicators and is appropriate for scales with a small number of items.^40^, ^41^ After selecting the best‐fitting CFA models, factor scores were calculated and correlated with summed trauma score, SAD, SP, GAD, OCD, PD, and MDD to determine the convergent validity of the ITQ‐CA.

In the second step, a latent profile analysis (LPA) was conducted, testing models with two to six latent classes. Model fit was assessed using information criterion statistics, the Lo‐Mendell‐Rubin adjusted likelihood ratio test (LMR‐A)^42^ and entropy. A non‐significant LMR‐A indicates that a model with one less class should be selected^43^ and higher entropy values indicated greater classification accuracy.^44^ ‘Elbow plots’ were used to determine the point at which there were diminished gains in model fit.^43^, ^45^In the third step, a series of FMMs were fit to the data. The number of factors from the best‐fitting CFA model were used for the FMM while number of classes from the best‐fitting LPA model were used as the upper‐limit for extracting classes in the FMM.^9^ Similar to a prior study,^10^ a variation of the ‘Type‐1’ FMMs was modelled where class‐specific item‐level intercepts were estimated rather than factor means.^9^ To avoid solutions based on local maxima, 500 random sets of starting values were initially used and 100 final stage optimizations. The best‐fitting model was selected using the same criteria as the LPA. Profile plots for each solution were also examined to determine whether the classes comprising each solution described theoretically plausible and described meaningful groups of individuals.^46^ Average posterior probabilities greater than 0.70 and class sizes greater than 5% also indicated acceptability of a latent class solution.^47^ A chi‐square test was used to examine the accuracy of latent class membership with respect to ICD‐11 diagnostic status (i.e., no diagnosis, PTSD, CPTSD). Adjusted standardised residuals ≥1.96 indicated a statistically significant difference between observed and expected counts.

The R3step auxiliary command^48^ was employed to determine predictors of class membership. The first analysis included all predictors and total trauma score while the second analysis included all predictors, the aggregate trauma categories and the three traumas which were included individually because of being sufficiently distinct from all others. Two models were included to predict class membership to allow the examination of the influence of total trauma exposure and the cumulative effects of specific trauma types separately. Statistically significant effects for the predictors were indicated if 1 was outside the 95% confidence intervals (CI's).^49^ Differences across the latent classes in terms of mean scores on the RCADS subscales were examined using the Bolck‐Croon‐Hagenaars Method (BCH method),^50^ which provides both a Wald chi‐square test and pairwise comparisons. All multivariate and distal outcome analyses for the FMM classes were replicated for the trauma‐exposed participants (*n* = 507) using multinomial logistic regressions and multiple one‐way ANOVAs, respectively (see Data S1 for a more detailed explanation). Analyses were estimated using robust maximum likelihood estimation (MLR) in Mplus version 8.2,^51^ which is appropriate when item indicators are ordinal with more than three categories as were the ITQ‐CA indicators.^52^, ^53^ Initial descriptive and diagnostic groups analyses were produced using SPSS v.27.

## 3 RESULTS

### 3.1 Descriptive statistics

Of the entire survey sample of 11–19‐year‐olds in NI (*N* = 1293), 4.9% (*n* = 63) met the ITQ requirements for diagnosis of either PTSD or CPTSD; the prevalence of PTSD was 1.5% (*n* = 19) and the prevalence of CPTSD was 3.4% (*n* = 44). There was no statistically significant gender differences for PTSD (males = 1.8%, females = 1.1%; $x^{2}\;$(1) = 1.10, *p* < 0.29) but there were significantly more females who met criteria for CPTSD (male = 2.3%, females = 4.6%; $x^{2}\;$(1) = 5.34, *p* < 0.05). The number of traumas reported by the survey sample ranged from 0 to 10, with an average of 0.78 (*SD* = 1.36; Median = 0.00) and more than a third of the survey sample (37.5%; *n* = 386) reported exposure to at least one trauma. The most common traumas were serious accident or injury (18.4%; *n* = 217), witnessing violence at school or in the community (19.7%; *n* = 220) and the sudden or violent death of a loved one (11.7%; *n* = 138). Additionally, 3.2% (*n* = 38) reported exposure to one sexual trauma and 1.5% (*n* = 18) reported two or more sexual traumas. Moreover, 17.9% (*n* = 211) reported exposure to one trauma involving direct harm or violence and 7.2% (*n* = 85) reported exposure to two or more traumas involving direct harm or violence. Finally, 17.9% (*n* = 211) reported exposure to one vicarious trauma and 4% (*n* = 47) reported exposure to two or more vicarious traumas.

### 3.2 FMM results

Table S1 reports the fit statistics for the CFA and shows that Model 1 and Model 4 had a poor fit. Model 2 ($x^{2}\;\left(39\right)=91.239,p<0.001,\text{RMSEA}=0.051,\mathrm{CFI}=0.978,\mathrm{TLI}=0.962;\mathrm{BIC}=15128.349)$ and Model 3 ($x^{2}\;\left(66\right)=120.695,p<0.001,\text{RMSEA}=0.056,\mathrm{CFI}=0.968,\mathrm{TLI}=0.955;\mathrm{BIC}=15126.877)$ demonstrated acceptable fit. Although the chi‐squared statistic was significant for both Model 2 and Model 3, neither model should be rejected based on this as the power of chi‐square tests is positively associated with sample size.^54^Model 2 and Model 3 were relatively similar in terms of fit, however, Model 3 was selected because of being most parsimonious and having the lowest BIC value. All indicators loaded significantly (*p* < 0.001) and strongly (>0.76) onto the first‐order PTSD and DSO latent factors and all first‐order factors loaded strongly onto their corresponding second‐order factors (all >0.85). The correlation between the DSO and PTSD latent factors was high (*r* = 0.79, *p* < 0.001). CR estimates were excellent for both the PTSD sub‐scale (CR = 0.83) and DSO sub‐scales (CR = 0.88). Standardised factor loadings for Model 3 are demonstrated in Table S2. Convergent validity of the ITQ‐CA was tested using factor scores derived from Model 3. The correlations between the first‐order factor scores and RCADS sub‐scales were all high, positive and statistically significant (see Table S3). There was a moderate association between total trauma score and PTSD (*r* = 0.352) and DSO (*r* = 0.332).

In terms of the LPA (Table 1), the LMR‐A was non‐significant (*p* < 0.05) for the five‐class solution, indicating that the four‐class solution should be selected. However, log‐likelihood and information criterion values failed to reach a minimum. Elbow plots demonstrated no meaningful improvements after the four‐class model while inspection of profile plots for the two‐class to four‐ class solutions showed how all classes in the four‐class model represented distinct and important trauma profiles. Inspection of the five‐class solution indicated that the additional class was a bisection of the CPTSD class identified in the four‐class solution and thus, the four‐class solution was retained as the optimal LPA model.

**TABLE 1:** Fit statistics for the CFA, LCA and FMM of ICD‐11 PTSD and CPTSD

Model	Log‐likelihood	AIC	BIC	ssaBIC	Entropy	LMR‐A (p)
CFA						
Model 1	−7765.417	15602.834	15755.061	15640.792	‐	‐
Model 2	−7405.347	14912.695	15128.349	14966.469	‐	‐
**Model 3**	**−7429.525**	**14945.051**	**15126.877**	**14990.390**	‐	‐
Model 4	−7514.687	15113.375	15290.972	15157.659		
LPA						
2 classes	−9414.979	18877.957	18979.442	18903.263	0.979	0.0000
3 classes	−8005.346	16084.692	16241.146	16123.704	0.942	0.0000
**4 classes**	**−7703.550**	**15507.100**	**15718.525**	**15559.819**	**0.947**	**0.0093**
5 classes	−7501.776	15129.532	15395.929	15195.959	0.940	0.3328
6 classes	−7396.109	14944.218	15265.585	15024.352	0.946	0.4006
FMM						
2 factors 2 classes	−7285.996	14683.991	14920.788	14743.037	0.935	0.0160
2 factors 3 classes	−7179.766	14497.532	14789.300	14570.286	0.942	0.2600
**2 factors 4 classes**	−7092.078	**14348.156**	**14694.893**	**14434.616**	**0.967**	0.4343
2 factors 5 classes	−7035.538	14261.077	14662.785	14361.244	0.963	0.4127

*Note*: Bold indicates best fitting models.

**FIGURE 1:**
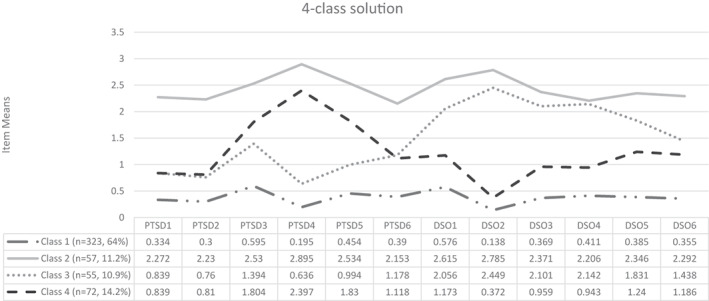
Profile plot of item‐level means for two‐factor four‐class factor mixture model solution

For the FMM analyses (Table 1), LMR‐A became non‐significant for the two‐factor three‐class solution, however, information criterion values (i.e., AIC, BIC, ssaBIC) continued to decrease with each additional class. Inspection of profile plots for the two‐, three‐ and four‐class solutions indicated that each additional class represented an important and meaningful trauma group. The four‐class solution identified an additional trauma group who differed both quantitatively and qualitatively from all other classes. Average posterior probabilities for the classes comprising the two‐factor four‐class FMM were high (>0.97), class sizes were adequate (> 10%) and classes were most consistent with the ICD‐11 description of PTSD and CPTSD. Furthermore, entropy was highest for the four‐class solution, indicating that the two‐factor four‐class solution resulted in improved classification certainty. Figure 1 illustrates the profile plot for the ITQ‐CA symptom endorsement patterns within each class. The largest class was Class 1 (64%, *n* = 323) which was labelled ‘low symptom endorsement class’ because of low endorsement of PTSD and DSO symptomology. Class 2 (11.2%, *n* = 57) was labelled ‘CPTSD class’ because of high endorsement of all PTSD and DSO symptom indicators. Class 3 (10.9%, *n* = 55) was labelled ‘DSO class’ because of low endorsement of the PTSD symptom indicators and high endorsement of the DSO items, in particular the AD and NSC items. Class 4 (14.2%, *n* = 72) was characterised by higher endorsement of both the PTSD and DSO items compared with ‘low symptom endorsement class’. In comparison to the ‘DSO class’, the endorsement of PTSD items, especially Av, was greater while endorsement of the DSO items was relatively smaller. This class was labelled ‘PTSD class’.

### 3.3 Agreement between ITQ classification and FMM classes

The chi‐square test of association (see Table S4) demonstrated a significant association between diagnostic status derived from the ITQ scoring and the FMM class membership, $x^{2}$(6, *N* = 507) = 189.189, *p* < 0.001. This effect was moderate (Cramer's V = 0.43).^43^ The degree of concordance between diagnostic status and membership of the ‘low symptom endorsement class’, ‘PTSD class’ and ‘CPTSD class’ was high (i.e., 69.8%; 354). Results showed how membership of the ‘low symptom endorsement class’ was associated with no diagnosis (adjusted standardised residual = 9.84), membership of the ‘CPTSD class’ was associated with both PTSD diagnosis (adjusted standardised residual = 4.3) and CPTSD diagnosis (adjusted standardised residual = 11.51), and ‘PTSD class’ membership was associated with PTSD diagnosis (adjusted standardised residual = 3.55). There was no statistically significant association between membership of the ‘DSO class’ and having no diagnosis (adjusted standardised residual = −0.94), a PTSD diagnosis (adjusted standardised residual = −0.80) or a CPTSD diagnosis (adjusted standardised residual = 1.64).

### 3.4 Multivariate regression predicting class membership

Two regression models were estimated, the first to examine total trauma exposure and the second to examine the cumulative effects of specific trauma types (i.e., sexual, violent and vicarious traumas). For the first multivariate analysis (see Table 2), older adolescents were at increased risk of membership of the ‘CPTSD class’ (OR = 1.230), ‘PTSD class’ (OR = 1.187) and ‘DSO class’ (OR = 1.158) compared to ‘low symptom endorsement class’. Higher area level deprivation increased risk of membership of the ‘DSO class’ (OR = 1.203). Participants who had a parent with high ACE scores were less likely to be members of the ‘PTSD class’ (OR = 0.453). Female participants were more likely to be members of ‘CPTSD class’ (OR = 3.327) and ‘PTSD class’ (OR = 1.867). Young people with SEN were at increased risk of membership of ‘CPTSD class’ (OR = 3.143). Higher levels of trauma significantly increased likelihood of membership of the ‘CPTSD class’ (OR = 1.587), ‘DSO class’ (OR = 1.511) and ‘PTSD class’ (OR = 1.377). For the second multivariate analysis where the cumulative effects of specific trauma types were examined (see Table 3), exposure to war (OR = 0.198) and natural disaster (OR = 0.128) were uniquely predictive of ‘CPTSD class’ membership, while living with both parents uniquely predicted ‘DSO class’ membership (OR = 0.551). Moreover, compared with participants with no sexual trauma exposure, participants in the ‘CPTSD class’ were more likely to report one sexual trauma (OR = 2.883) and two or more sexual traumas (OR = 22.594). Participants with two or more experiences of direct harm or interpersonal threat were over seven times more likely to be members of ‘CPTSD class’ (OR = 7.128) and over three times more likely to be members of the ‘partial‐PTSD class’ (OR = 3.467). Participants in the ‘DSO class’ were more likely to report one sexual trauma (OR = 2.823) and were over eight times more likely to report two or more vicarious traumas (OR = 8.353).

**TABLE 2:** Demographic and trauma‐related predictors (total trauma score) and latent class membership (adjusted odds ratios)

Predictor	Class 2: CPTSD OR (95% CI)		Class 3: DSO OR (95% CI)		Class 4: Partial‐PTSD OR (95% CI)	
Parent GHQ (caseness)	1.176	(0.520, 2.658)	0.754	(0.313, 1.818)	1.670	(0.869, 3.208)
Out‐of‐home care	1.284	(0.412, 4.007)	0.324	(0.022, 4.782)	1.212	(0.400, 3.676)
Special education needs	3.143*	(1.318, 7.495)	0.520	(0.126, 2.150)	1.826	(0.791, 4.215)
Parent ACE (≥4)	1.087	(0.440, 2.686)	1.219	(0.440, 3.375)	0.453*	(0.152, 1.355)
Age	1.230*	(1.067, 1.417)	1.158*	(1.012, 1.324)	1.187**	(1.056, 1.335)
Living with both parents	0.667	(0.320, 1.387)	0.620	(0.314, 1.222)	0.933	(0.510, 1.707)
Family in receipt of social welfare	0.904	(0.419, 1.954)	1.435	(0.685, 3.003)	0.846	(0.454, 1.578)
Gender (female)	3.327*	(1.671, 6.624)	1.319	(0.700, 2.486)	1.867*	(1.065, 3.273)
MDM decile (area level deprivation)	0.957	(0.845, 1.084)	1.203**	(1.070, 1.353)	0.948	(0.858, 1.047)
Total trauma score	1.587**	(1.287, 1.956)	1.511**	(1.217, 1.876)	1.377**	(1.146, 1.655)

*Note*: Class 1 (Baseline) is the reference category. *Significant at *p* < 0.05, **significant at *p* < 0.01.

Abbreviations: ACE, adverse childhood experiences; GHQ, general health questionnaire (i.e., mental health); MDM decile, multiple deprivation decile (i.e., area‐level deprivation).

**TABLE 3:** Demographic and trauma‐related predictors (aggregate trauma domains) and latent class membership (adjusted odds ratio)

Predictors	Class 2: CPTSD OR (95% CI)		Class 3: DSO OR (95% CI)		Class 4: Partial‐PTSDOR (95% CI)	
Parent GHQ	1.189	(0.527, 2.682)	0.730	(0.266, 2.002)	1.790	(0.919, 3.485)
Out‐of‐home care	0.828	(0.224, 3.067)	0.397	(0.034, 4.641)	1.203	(0.419, 3.450)
Special education needs	3.697*	(1.532, 8.922)	0.528	(0.126, 2.207)	1.783	(0.743, 4.278)
Parent ACE	1.087	(0.421, 2.806)	0.950	(0.307, 2.943)	0.415	(0.120, 1.433)
Age	1.200*	(1.036, 1.390)	1.126	(0.979, 1.296)	1.203*	(1.061, 1.363)
Living with both biological parents	0.652	(0.286, 1.485)	0.551*	(0.272, 1.116)	0.927	(0.497, 1.730)
Family in receipt of social welfare	0.906	(0.379, 2.165)	1.218	(0.550, 2.694)	0.895	(0.448, 1.786)
Gender	3.171*	(1.459, 6.889)	1.283	(0.619, 2.662)	2.200*	(1.204, 4.019)
MDM Decile (area level deprivation)	0.920	(0.788, 1.073)	1.230**	(1.071, 1.414)	0.951	(0.852, 1.061)
Natural disaster	0.128**	(0.008, 2.018)	2.078	(0.486, 8.892)	1.246	(0.221, 7.016)
Stressful or scary medical procedure	1.014	(0.336, 3.057)	0.607	(0.155, 2.374)	1.378	(0.566, 3.353)
War	0.198**	(0.013, 3.122)	0.991	(0.056, 17.438)	2.206	(0.256, 18.999)
One sexual trauma^a^	2.883	(1.001, 8.303)	2.823	(1.028, 7.756)	0.463	(0.108, 1.980)
2 or more sexual traumas^a^	22.594*	(3.316, 153.932)	1.039	(0.026, 41.353)	0.547	(0.028, 10.856)
One violent trauma^b^	1.961	(0.916, 4.198)	1.261	(0.596, 2.668)	1.371	(0.705, 2.668)
Two or more violent trauma^b^	7.128*	(2.351, 21.616)	2.379	(0.872, 6.488)	3.467*	(1.399, 8.591)
One vicarious trauma^c^	0.565	(0.258, 1.240)	0.940	(0.430, 2.055)	1.522	(0.807, 2.871)
Two or more vicarious^c^	0.997	(0.247, 4.033)	8.353*	(3.033, 23.561)	1.873	(0.564, 6.223)

Abbreviations: ACE, adverse childhood experiences; MDM decile, multiple deprivation measure (i.e., area‐level deprivation); Parent GHQ, general health questionnaire (mental health).

^a^ compared to no sexual trauma.

^b^ compared to no violent trauma.

^c^ compared to no vicarious trauma.

### 3.5 Mental health differences across classes

Pairwise comparisons between classes (Table 4) using $x^{2}\;$ showed that SAD, SOC, OCD, PD, MDD and GAD scores were significantly higher for all classes compared with ‘low symptom endorsement class’. Moreover, pairwise comparisons between classes using $x^{2}\;$ showed that average SAD, SOC, OCD, PD, GAD, and MDD scores were significantly higher for the ‘CPTSD class’ compared with both the ‘partial‐PTSD class’ and ‘DSO class’. Average OCD, GAD and MDD scores were significantly higher for the ‘DSO class’ than the ‘partial‐PTSD class’. There were no significant differences in average SAD, SOC and PD scores for the ‘partial‐PTSD class’ and ‘DSO class’.

**TABLE 4:** Equality test of means of PHQ‐8, GAD‐7 and WHO‐5 scores across the latent classes

	Total sample	Class 1: Low symptom endorsement	Class 2: CPTSD	Class 3:DSO	Class 4: Partial‐PTSD	Overall Chi‐Square test	Pairwise comparison (*p* < 0.05)
	Mean (*SE*)	Mean (*SE*)	Mean (*SE*)	Mean (*SE*)	Mean (*SE*)		
Separation anxiety disorder (SAD)	3.300 (3.685)	2.241 (0.143)	7.913 (0.680)	3.817 (0.526)	3.998 (0.446)	83.816 (*p* < 0.001)	2, 3, 4 > 1 2 > 3,4
Social anxiety disorder (SOC)	11.708 (6.631)	9.821 (0.327)	17.650 (0.875)	14.483 (0.976)	13.312 (0.701)	93.732 (*p* < 0.001)	2, 3, 4 > 1 2 > 3, 4
Obsessive compulsive disorder (OCD)	4.435 (3.873)	3.055 (0.162)	9.160 (0.597)	6.581 (0.522)	5.226 (0.426)	145.030 (*p* < 0.001)	2, 3, 4 < 1 2 > 3,4, 3 > 4
Panic disorder (PD)	5.889 (6.090)	3.704 (0.235)	14.200 (0.989)	8.675 (0.754)	6.940 (0.729)	151.370 (*p* < 0.001)	2, 3, 4 > 1 2 > 3, 4
Generalised anxiety disorder (GAD)	8.452 (6.288)	4.528 (0.188)	10.578 (0.581)	8.818 (0.635)	6.840 (0.491)	141.709 (*p* < 0.001)	2, 3, 4 > 1 2 > 3, 4, 3 > 4
Major depressive disorder (MDD)	6.005 (4.304)	5.819 (0.238)	17.312 (0.830)	13.258 (0.869)	9.469 (0.641)	249.905 (*p* < 0.001)	2, 3, 4 > 1 2 > 3, 4, 3 > 4

### 3.6 Multivariate regression predicting ITQ caseness for trauma‐exposed sub‐set ( *n* = 507)

For the first multivariate regression (Table S5), female participants were more likely to meet criteria for CPTSD (OR = 3.249; C.I. = 2.048, 8.361), while higher cumulative trauma significantly increased likelihood of being in the CPTSD group (OR = 1.503; C.I. = 1.357, 2.070) and PTSD group (OR = 1.295; C.I. = 1.040, 1.612). For the second multinomial logistic regression (Table S6), individuals meeting criteria for CPTSD were more likely to be female (OR = 3.208; C.I. = 1.486, 6.928), to report one sexual trauma (OR = 4.108; C.I. = 1.466, 11.512), and to report two or more sexual traumas (OR = 15.595; C.I. = 4.050, 60.058). Participants who reported exposure to two or more traumas involving direct harm or violence were more likely to meet criteria for CPTSD (OR = 3.968; C.I. = 1.301, 12.096), and PTSD (OR = 5.062; C.I. = 1.113, 23.022).

Average scores on all RCADS subscales were highest for the CPTSD group, followed by the PTSD group and then the no diagnosis group (see Table S7). One‐way analysis of variance (ANOVA) results indicated that groups differed in terms of average levels of SAD (F[2, 504] = 42.06, *p* < 0.001, ω2 = 0.14), SOC (F[2, 504] = 43.07, ω2 = 0.14), OCD (F [2, 503] = 62.29, *p* < 0.001, ω2 = 0.20), PD(F [2, 503] = 80.42, *p* < 0.001, ω2 = 0.24), GAD (F [2, 504] = 57.40, *p* < 0.001, ω2 = 0.24) and MDD (F [2, 503] = 81.99, *p* < 0.001, ω2 = 0.18). Post hoc comparisons using the Tukey HSD test demonstrated that the average SAD, SOC, OCD, PD, MDD and GAD scores were significantly higher for the CPTSD group compared with the PTSD group. Average SAD and SOC scores were significantly higher for the CPTSD group compared with the PTSD and no diagnosis groups, however these was no statistically significant difference between the PTSD and no diagnosis group. Compared with the no diagnosis group, average OCD, PD, GAD and MDD scores were significantly higher for the PTSD and CPTSD groups.

## 4 DISCUSSION

The primary objectives of the current study were to determine the prevalence, construct validity, risk factors and psychological correlates associated with ICD‐11 PTSD and CPTSD in a representative general‐population sample of young people from NI. Consistent with prior research on adult populations,^10^, ^55^ a hybrid model which captured the quantitative and qualitative distinction between PTSD and CPTSD was best‐fitting. Similar to findings from the Austrian foster children study,^7^ a two‐factor second‐order model was deemed the best dimensional representation of the ITQ‐CA, although the correlated six‐factor model provided equally close fit to the data. These findings mirror the adult literature^5^ and show that the distinctive nature of PTSD and DSO symptoms can be captured at the first and second‐order level in both youth and adult samples. For the chosen model, all ITQ‐CA indicators loaded strongly onto their constituent dimensions while CR estimates supported the reliability of the ITQ‐CA. The correlation between the higher‐order PTSD and DSO factors was high (i.e., *r* = 0.79), however was significantly lower than observed in a prior study using the ITQ‐CA^7^ and was largely similar prior studies using the ITQ.^21^, ^56^, ^57^ Consistent with findings from a prior study,^7^ the ITQ‐CA demonstrated convergent validity through strong associations with total trauma exposure and psychopathological outcomes.

The identification of trauma groups reflecting the distinction between PTSD and CPTSD concurs with the ICD‐11 description of trauma‐based psychopathology,^57^ while the presence of a ‘DSO class’ adds to the growing number of general population studies in which this profile has also been identified.^6^, ^10^, ^18^, ^58^ It should be noted however, that a ‘pure’ PTSD class was not identified but rather a ‘Partial‐PTSD class’ whereby endorsement of the avoidance and sense of threat items were elevated but endorsement of the re‐experiencing items was low. Some prior research has demonstrated low endorsement of the re‐experiencing cluster in young people^59^ and thus, monitoring of the performance of this symptom cluster across future studies of young people is necessary. Nevertheless, the re‐experiencing items were strongly endorsed by the ‘CPTSD class’, and thus, it may be that given the lower prevalence of PTSD in this sample, that this class was also likely capturing individuals with symptom profiles which did not capture the full spectrum of required symptoms for ICD‐11 PTSD.

Collectively, the superiority of the hybrid (i.e., FMM) model provides further support for the validity of conceptualisation of ICD‐11 PTSD and CPTSD as representing distinct diagnostic entities which differ both quantitatively and qualitatively. These findings contradict those of a prior study which also utilised FMM to investigate the latent structure of PTSD and CPTSD using proxy items^60^ and found that although a FMM was found to best capture the latent structure of PTSD and CPTSD, the identified classes differed only in respect to symptom severity levels rather than disorder type. This led those authors to challenge the validity of the ICD‐11 PTSD and CPTSD distinction. Supporting prior findings,^10^ the degree of concordance between FMM class membership and diagnostic status as determined by the ITQ‐CA diagnostic algorithm was high. No association was evident between ‘DSO class’ membership and PTSD or CPTSD diagnostic status, suggesting that this class is not erroneously capturing probable PTSD or CPTSD cases and that the ITQ‐CA is operating in its intended manner. Interestingly, there were fewer participants with no diagnosis in the ‘DSO class’ than expected, indicating that this class is capturing individuals with a symptom profile distinct from those who are non‐symptomatic. Although it has been suggested that PTSD may be more common in community samples and CPTSD in clinical samples,^61^ the prevalence of CPTSD was found to exceed that of PTSD in the present study. This finding corresponds with several other general population studies,^3^, ^19^, ^20^ suggesting that CPTSD may be at least as common as PTSD in the general population when assessed by self‐report measures. The developmental stage of participants in the present study may explain why CPTSD was more prevalent such that early developmental trauma can lead to more pervasive difficulties across multiple domains including affective regulation, self‐perceptions, and relationships.^62^ Conversely, the pervasive psychological and social effects from ‘the Troubles’ may cultivate a developmental environment where propensity for trauma and complex posttraumatic responses are heightened.

Inconsistent with findings from a prior study,^23^ older age rather than younger age predicted membership of all trauma‐response profiles compared with the reference class. The greater propensity for trauma exposure and engagement in high‐risk behaviours during later adolescence likely increases the potential for stress‐related disorders.^63^ Consistent with prior research,^7^, ^21^, ^23^ females were more likely to be members of the ‘partial‐PTSD class’ and ‘CPTSD class’. Factors such as trauma type,^64^, ^65^, ^66^ peritrauma and posttrauma factors^67^ and differences in hormonal and biological stress response systems,^68^ have been proposed as explanations for such gender differences in post‐traumatic distress. SEN was identified as a unique risk factor for ‘CPTSD class’ membership, underscoring the importance of social factors in the aetiology of CPTSD in young people.^17^ It may be that young people with compromised intellectual functioning are more vulnerable to trauma exposure (e.g., being a victim of violence), may have fewer protective factors (e.g., social support) and/or are less adept to adaptively navigate traumatic aftermath.^63^ Common features of SEN including peer, social and emotional difficulties which may act as factors which increase risk and/or reduce protection against trauma exposure and the development of CPTSD symptoms. For instance, peer, social and emotional difficulties are risk‐factors for deployment of expressive suppression strategies in young people,^69^ an emotion regulation strategy which has been observed in individuals with CPTSD.^70^ Low levels of perceived social support have also been identified as a risk‐factor for CPTSD.^71^ Further research is necessary to disentangle the mechanisms underpinning this association.

Consistent with prior research,^7^, ^19^, ^21^ a clear dose–response association was evident between cumulative trauma and membership to all trauma classes, and this effect was strongest for the ‘CPTSD class’. Consistent with prior studies where sexual trauma was identified as a pre‐dominant risk factor for CPTSD,^19^, ^21^, ^72^, ^73^ experiences of sexual trauma at lower and higher frequencies of exposure significantly predicted ‘CPTSD class’ membership. Notably, sexual trauma was also a risk‐factor for ‘DSO class's membership,^74^ although this effect was applicable only at lower quantities of exposure. It is likely that DSO symptoms resonate strongly with victims of sexual trauma. For instance, affective dysregulation is common for young people exposed to a sexual trauma,^75^ while low self‐esteem and feelings of shame and guilt are also common.^76^, ^77^, ^78^, ^79^ It is possible that at lower quantities of exposure some young people are more vulnerable to the DSO symptoms rather than the core PTSD symptoms. Moreover, because (1) DSO symptoms are cross‐diagnostic,^18^ and (2) sexual trauma is linked to various other forms of psychopathology including depression, anxiety, disordered eating, and substance abuse disorders,^80^ it may be that this class captures trauma‐exposed individuals with other psychological disorders.^23^, ^81^

In accord with prior studies,^6^, ^72^ exposure to traumatic events involving direct harm or violence was a risk factor for ‘partial‐PTSD class’ and ‘CPTSD class’ membership, but only observable at the upper threshold of exposure (i.e., ≥2 exposures). Because exposure to a violent traumatic event at one time‐point has been shown to predict PTSD following re‐exposure at subsequent time‐points,^82^ it may be that the combining and cumulative effects of exposure to such traumatic events is the key determinant of post‐traumatic psychopathology. Exposure to higher quantities of vicarious trauma was identified as a unique risk factor of ‘DSO class’ membership. Prior research has shown how although vicarious trauma increases risk for psychopathology, the probability of maladaptive psychological outcomes, especially PTSD, is considerably lower than direct exposure.^82^, ^83^ Further research is required to determine whether such findings replicate in other samples.

Supporting the idea that CPTSD is a more comorbid and debilitating condition,^20^, ^84^, ^85^, ^86^, ^87^ young people in the ‘CPTSD class’ reported elevated anxiety and depression symptomology compared with all other classes. Overall, these results suggest that it is not only trauma type but also quantity of trauma exposure which confers greater vulnerability for posttraumatic psychopathology. Confirming observations from a prior study,^21^ patterns of comorbidity appear to differ in young people. Specifically, prior research on adults has indicated depressive symptoms to be more closely related to DSO, and anxiety symptoms to PTSD,^88^ whereas average levels of anxiety and depressive symptoms were significantly higher for the ‘DSO class’ compared with the ‘partial‐PTSD class’ in the current study. Moreover, similar to prior studies, the ‘DSO class’ reported higher levels of depression and anxiety than the ‘partial‐PTSD class’.^10^, ^23^ Affective dysregulation,^89^, ^90^ interpersonal problems^91^, ^92^ and negative self‐concept^93^, ^94^ are predominant features of many psychological disorders, and thus the high degree of comorbidity between ‘DSO class’ membership and other forms of psychopathology may indicate that this class does indeed represent trauma‐exposed young people whose symptom profile is better described by an alternative psychiatric diagnosis.

To ensure the validity of our findings surrounding risk‐factors and psychological outcomes associated with membership of both the ‘partial‐PTSD class’ and ‘CPTSD class’, all analyses were replicated for the ITQ‐CA diagnostic groups, with the majority of findings replicating. Notably, females were identified as being at greater risk for diagnosis of CPTSD only however, female gender was a significant risk‐factor for ‘partial‐PTSD class’ membership in the FMM analyses. The higher prevalence of PTSD in males in the present study may explain this finding which is likely because of the fact that (1) males reported significantly higher levels of exposure to experiences of direct harm or violence exposure, and (2) previous research has shown violence exposure to be a significant risk‐factor for the development of PTSD.^95^, ^96^ It is possible that although females are more likely to present with symptom patterns consistent with PTSD, males are more likely to meet the formal diagnostic requirements. Likewise, the discrepant findings regarding the role of gender in predicting membership of the FMM classes and the diagnostic groups themselves may be partially explained by the small proportion of participants who met criteria for diagnosis of PTSD and thus, predictors of FMM class membership are not solely targeting those diagnostic cases but rather a broader spectrum of participants with symptom endorsement patterns consistent with PTSD. Two or more traumas involving direct harm or violence predicted PTSD diagnosis to a greater extent than CPTSD diagnostic status while the reverse was true for the FMM classes. Such minor alterations in magnitudes of effects are to be expected when using the more stringent diagnostic criteria and also given the small proportion of participants who met criteria for diagnosis of PTSD and CPTSD. Nonetheless, these findings indicate cumulative exposure to traumatic events of a violent nature to be a salient risk‐factor for diagnosis of both PTSD and CPTSD.

There are several notable strengths of the current study including that this is the first study of its' kind to investigate the prevalence, latent structure, correlates and co‐morbidities associated with PTSD and CPTSD in young people from NI. Nonetheless, this study has some limitations. First, only 12.9% (*n* = 64) of participants in the present study were in contact with a mental health specialist and therefore, future research should attempt to replicate the methodological procedure adopted in the current study in samples of young people in receipt of clinical support. Second, anxiety and depression were the only psychological outcomes assessed in the current study because of the abundance of literature indicating high levels of co‐occurrence among these disorders.^18^, ^20^, ^78^, ^79^, ^84^, ^85^, ^86^, ^87^ However, other disorders associated with both disorders include dissociation^7^, ^18^, ^97^ and borderline personality disorders,^98^, ^99^ both of which were not assessed as part of the NI‐YWS. Third, the use of a self‐report measure to assess childhood trauma was a limitation of the current study given that under‐reporting of childhood trauma is common,^100^ especially for young people who may be fearful of disclosing specific traumas.^101^ Moreover, all other measures utilised in the present study were also self‐report including the ITQ‐CA and thus, replication of this study is required using clinician administered measures such as the International Trauma Interview,^102^ a semi‐structure interview designed to assess PTSD and CPTSD. Finally, the cross‐sectional nature of this study limits inferences regarding causality.

In conclusion, this study is first to demonstrate support for the validity of ICD‐11 PTSD and CPTSD as measured by the ITQ‐CA in a representative general‐population sample of young people from NI through the application of FMM. Novel findings regarding risk‐factors associated with PTSD and CPTSD will contribute towards the formulation of targeted guidelines for the assessment, treatment and prevention of these conditions, in particular for CPTSD where the evidence base is only beginning to flourish. There is now an urgent need to develop effective interventions for CPTSD in children and young people.

## AUTHOR CONTRIBUTIONS

Enya Redican and Mark Shevlin developed the study protocol and completed the analyses. Enya Redican drafted the paper. All authors provided critical revisions and approved the final version of the manuscript.

## CONFLICT OF INTEREST

Dr Marylene Cloitre was a member of the World Health Organisation Working Group on the classification of disorders associated with stress, reporting to the International Advisory Group for the revision of ICD‐11 Mental and Behavioural Disorders. Professor Mark Shevlin, Professor Philip Hyland and Professor Thanos Karatzias were part of the International Trauma Questionnaire development team and authors on the ITQ validation paper. The views provided in this article are the opinions of the authors and do not represent WHO policy.

## Supporting information


**Appendix S1** Supporting Information.

## Data Availability

Research data are not shared.
