# Supplementary material for: Prevalence and predictors of ICD‐11 posttraumatic stress disorder and complex PTSD in young people
Source: Acta Psychiatr Scand. 2022 May 11;146(2):110–25. doi: 10.1111/acps.13442 (PMC9540630; doi:10.1111/acps.13442)
Supplement: Supplementary file 1 — Appendix S1 Supporting Information. [file ACPS-146-110-s001.docx]

**Supplementary Materials**

**Additional information on trauma items included in the CATs.**

The CATS trauma screener comprised of 13-items including exposure to (1) natural disaster, (2) serious accident or injury, (3) threatened, hit or hurt badly in family, (4) threatened, hit or hurt badly in school or the community, (5) attacked, stabbed, shot at or robbed by threat, (6) seeing someone in family threatened, hit or hurt badly, (7) seeing someone in school or community threatened, hit or hurt badly, (8) sexual assault, (9) sexual molestation, (10) online sexual harassment, (11) sudden or violent death of a close one, (12) stressful or scary medical procedure, and (13) being around war.

**Additional information on FMM**

There are multiple different formulations of the FMM which differ with respect to levels of restriction and interpretation. Similar to a prior study^10^, a variation of the ‘type-1’ FMM was employed. ‘Type-1’ FMMs are generally characterised by class-varying factor means, class-invariant item intercepts, factor loadings, and a factor covariance matrix fixed at zero^9^. However, rather than allowing factor means to vary, the current study estimated class-specific item-level intercepts which allowed for the examination of the relative important of individual ITQ-CA symptom indicators for each of the latent classes. Factor means were fixed at zero to achieve model identification. This model suggests that differences across classes are the result of different endorsement of ITQ-CA indicators with it being the onus of the research to determine whether these classes differ qualitatively^44^.

**Description of aggregate trauma categories**

Due to the low endorsement of many of the traumas types (e.g. sexual assault was endorsed by 3.9% (n=20), sexual molestation was endorsed by 4.9% (n=25), and being attacked, stabbed or robbed by threat was also endorsed by 3.7% (n=19) of the sample and to retain power, several aggregate trauma categories were created consisting of traumas of a similar nature. These categories included (1) sexual trauma (i.e., sexual assault, sexual molestation and online sexual harassment), (2) exposure to direct harm or violence (i.e., serious accident or injury, victim of violence in the home or in school/community, attacked, stabbed, shot at or robbed by threat), and (3) vicarious violence exposure (i.e., witnessing violence at home or in the school/community). Due to dissimilarities with other traumatic stressors, three traumas (i.e., war, natural disaster, medical procedure) were included individually for completeness.

**Further information on ANOVA**

For the ANOVAs, omega-squared (ω^2^) was selected to quantify the magnitude of effects, while Tukey HSD test was used for post-hoc comparisons. Omega-squared is interpreted in the same way as Cohen’s *d* with 0.10 considered as a small effect, 0.30 considered as a moderate effect and 0.50 considered as a strong effect (Cohen, 1988)^53^. Standardised differences between means for each of pairwise comparison were calculated using Cohen’s *d*.

**
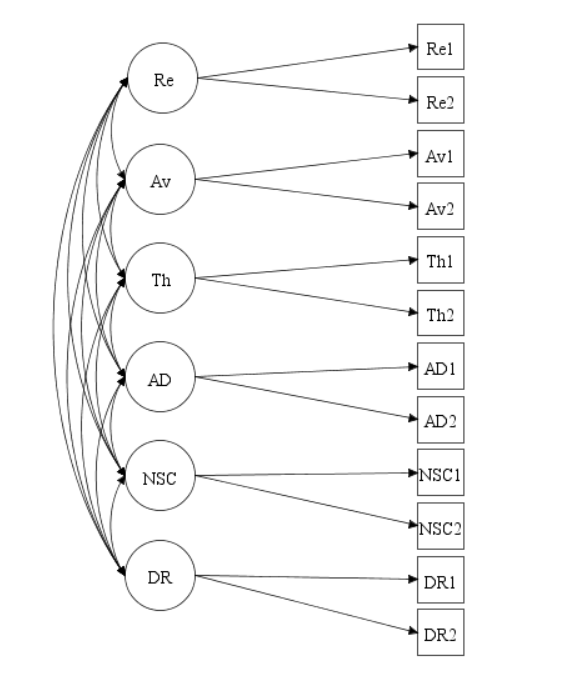

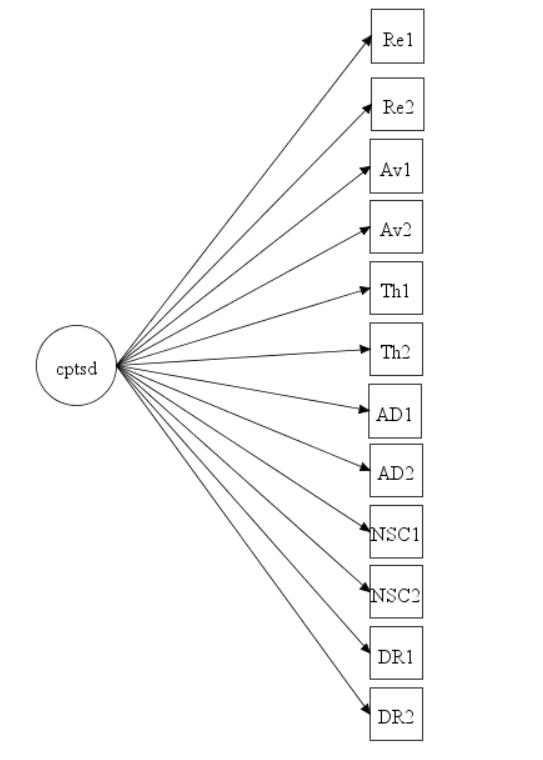
**

**Figure 1.B.** Model 2

Figure 1.A. Model 1

.

**
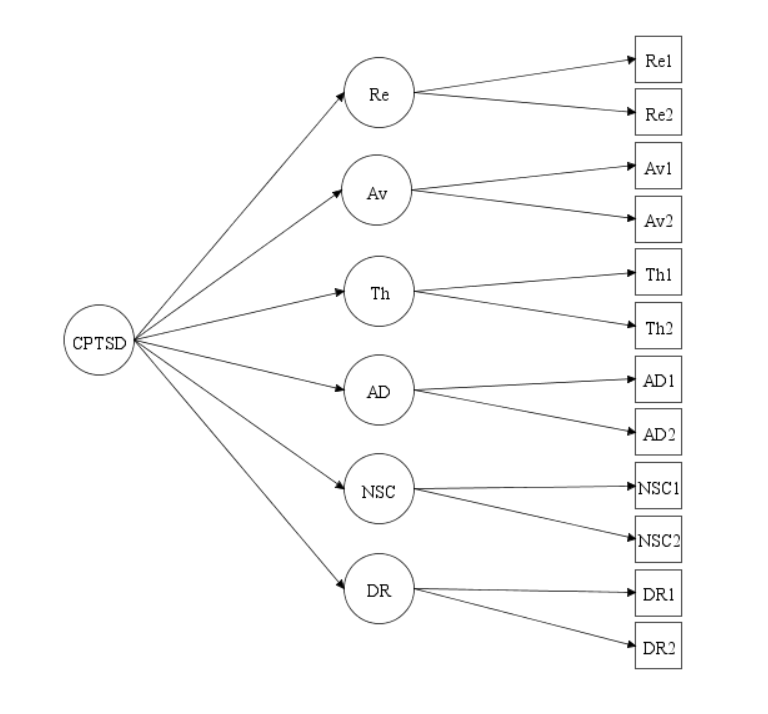

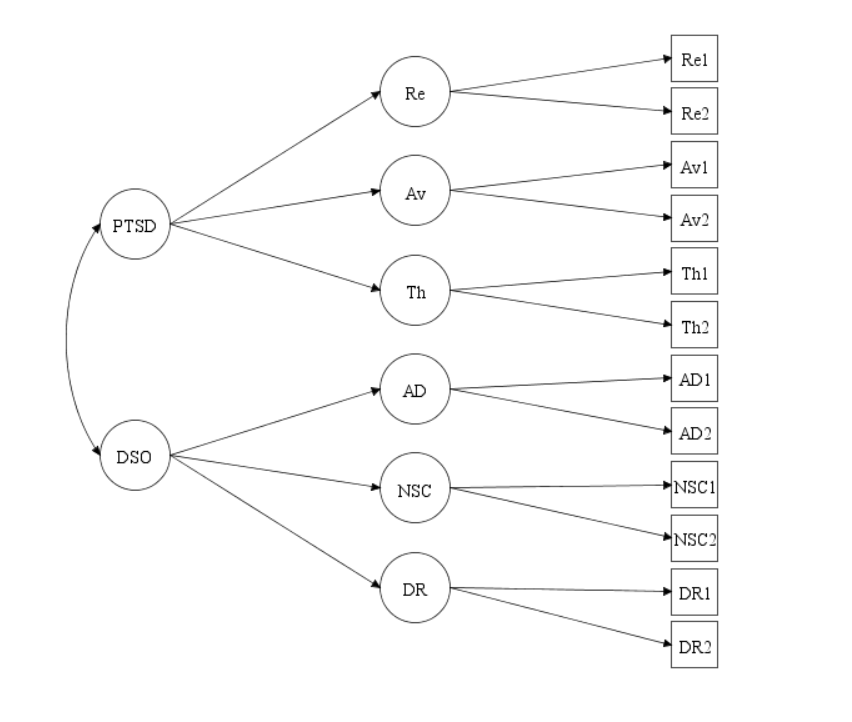
**

Figure 1.C.: Model 3

Figure 1.D: Model 4

**Supplementary Table 1: Fit statistics for two-factor second-order confirmatory factor analysis (CFA) model.**

| Model | Chi-square (df) | AIC | BIC | ssaBIC | CFI | TLI | RMSEA (95% C.I.) | SRMR |
| --- | --- | --- | --- | --- | --- | --- | --- | --- |
| Model 1 | 519.269 (54) * | 15602.834 | 15755.061 | 15640.792 | 0.800 | 0.755 | 0.130 (0.120, 0.141) | 0.073 |
| Model 2 | 91.239 (39)* | 14912.695 | 15128.349 | 14966.469 | 0.978 | 0.962 | 0.051 (0.038, 0.065) | 0.024 |
| Model 3 | **120.695 (47) *** | **14945.051** | **15126.877** | **14990.390** | **0.968** | **0.955** | **0.056 (0.043, 0.068)** | **0.033** |
| Model 4 | 226.490 (48)* | 15113.375 | 15290.972 | 15157.659 | 0.923 | 0.894 | 0.086 (0.075, 0.097) | 0.056 |

*Note: **p >.05; df= degrees of freedom; AIC= Akaike Information criterion; BIC= Bayesian Information Criterion; ssaBIC = sample-size adjusted BIC; CFI= comparative fit index; TLI = Tucker-Lewis Index; RMSEA= root mean square error of approximation; SRMR= standardised root mean residual.

**Supplementary Table 2:** Standardized factor loadings and standard errors for the two-factor second-order CFA.

|  | Re | Av | Th | AD | NSC | DR |
| --- | --- | --- | --- | --- | --- | --- |
| “Bad dreams reminding me of what happened”. | .833 (.027) |  |  |  |  |  |
| “Pictures in my head of what happened”. | .824 (.029) |  |  |  |  |  |
| “Trying not to think about what happened. Or not have feelings about it”. |  | .769 (.031) |  |  |  |  |
| “Staying away from anything that reminds me of what happened”. |  | .776 (.030) |  |  |  |  |
| “Being overly careful”. |  |  | .823 (.028) |  |  |  |
| “Being jumpy”. |  |  | .764 (.035) |  |  |  |
| “Having trouble calming down when upset”. |  |  |  | .772 (.027) |  |  |
| “Not being able to have any feelings or feeling empty inside”. |  |  |  | .815 (.027) |  |  |
| “Feeling like a failure”. |  |  |  |  | .902 (.021) |  |
| “Thinking I am not a good person”. |  |  |  |  | .861 (.027) |  |
| “Not feeling close to people”. |  |  |  |  |  | .932 (.016) |
| “Having a hard time staying close to other people”. |  |  |  |  |  | .890 (.020) |
| **Second-order factor loadings** |  | PTSD |  |  | DSO |  |
| Re-experiencing (Re) |  | .903 (.027) |  |  |  |  |
| Avoidance (Av) |  | .974 (.029) |  |  |  |  |
| Sense of threat (Th) |  | .888 (.034) |  |  |  |  |
| Affective dysregulation (AD) |  |  |  |  | 1.010 (.026) |  |
| Negative Self-concept (NSC) |  |  |  |  | .913 (.023) |  |
| Disturbances in relationships (DR) |  |  |  |  | .853 (.029) |  |

| **Factor Scores** | **Trauma** | **GAD** | **MDD** | **PD** | **OCD** | **SOC** | **SAD** |
| --- | --- | --- | --- | --- | --- | --- | --- |
| Re | .356 | .591 | .632 | .644 | .618 | .491 | .528 |
| Av | .344 | .618 | .672 | .666 | .640 | .535 | .555 |
| Th | .333 | .619 | .658 | .675 | .636 | .529 | .591 |
| AD | .334 | .686 | .791 | .712 | .672 | .614 | .569 |
| DR | .312 | .595 | .755 | .620 | .611 | .550 | .506 |
| NSC | .304 | .680 | .726 | .683 | .636 | .628 | .537 |
| PTSD | .352 | .633 | .688 | .683 | .653 | .545 | .570 |
| DSO | .332 | .684 | .792 | .712 | .670 | .609 | .568 |

**Supplementary Table 3:** Convergent validity of the ITQ-CA

GAD= generalised anxiety disorder, MDD= major depressive disorder, PD = panic disorder, OCD= obsessive compulsive disorder, SOC = social anxiety disorder, SAD = separation anxiety disorder

All correlations are significant at p<.001.

**Supplementary Table 4:** Crosstabulations of Class membership by diagnosis type.

| Class  Diagnostic group | Count | Expected Count | % within diagnostic group | Adjusted Standardised Residual |
| --- | --- | --- | --- | --- |
| Class 1 (low symptoms) | | | | |
| No symptoms | 318 | 282.9 | 71.6% | 13.0 |
| PTSD | 2 | 12.1 | 10.5% | -4.9 |
| CPTSD | 3 | 28 | 6.8% | -8.2 |
| Class 2 (CPTSD class) | | | | |
| No symptoms | 21 | 49.9 | 4.7% | -12.3 |
| PTSD | 8 | 2.1 | 42.1% | 4.3 |
| CPTSD | 28 | 4.9 | 63.6% | 11.5 |
| Class 3 (DSO class) | | | | |
| No symptoms | 46 | 48.2 | 10.4% | -0.9 |
| PTSD | 1 | 2.1 | 5.3% | -0.8 |
| CPTSD | 8 | 4.8 | 18.2% | 1.6 |
| Class 4 (PTSD class) | | | | |
| No symptoms | 59 | 63.1 | 13.3 | -1.6 |
| PTSD | 8 | 2.7 | 42.1 | 3.6 |
| CPTSD | 5 | 6.2 | 11.4 | -0.6 |

**Supplementary Table 5: Demographic and trauma-related (cumulative trauma) predictors of diagnostic status**

| Predictor | CPTSD diagnostic group (N=44)  OR (95% CI) | | PTSD diagnostic group (N=19)  OR (95% CI). | |
| --- | --- | --- | --- | --- |
| Parental ACEs | 0.862 | (0.272, 2.261) | 0.654 | (0.131, 3.268) |
| Special educational needs | 1.563 | (0.678, 4.627) | 3.299 | (0.998, 10.904) |
| Family in receipt of social welfare | 1.537 | (0.678, 3.211) | 1.029 | (0.305, 3.470) |
| MDM decile | 1.049 | (0.925, 1.190) | 0.974 | (0.816, 1.163) |
| Out-of-home care | 2.121 | (0.704, 5.047) | 2.867 | (0.761, 10.802) |
| Age | 1.077 | (0.958, 1.251) | 1.138 | (0.922, 1.406) |
| Parent GHQ score | 1.510 | (0.678, 2.840) | 1.757 | (0.568, 5.440) |
| Household Composition | 1.150 | (0.549, 2.195) | 0.668 | (0.212, 2.102) |
| Gender | 3.249* | (2.048, 8.361) | 0.853 | (0.336, 2.166) |
| Total Trauma | 1.503* | (1.357, 2.070) | 1.295* | (1.040, 1.612) |

Note: The ‘no diagnosis’ group is the reference category.

| **Supplementary Table 6: Demographic and trauma-related (trauma categories) predictors of diagnostic status.** | | | | |
| --- | --- | --- | --- | --- |
| Predictor | CPTSD diagnostic group  OR (95% CI) | | PTSD diagnostic group  OR (95% CI) | |
| Parent GHQ | 1.443 | (0.692, 3.009) | 2.165 | (0.648, 7.236) |
| Out-of-home care | 1.582 | (0.538, 3.651) | 3.326 | (0.801, 13.811) |
| Special education needs | 1.553 | (0.577, 4.180) | 3.348 | (0.992, 11.301) |
| Parent ACE | 0.727 | (0.213, 2.487) | 0.479 | (0.082, 2.805) |
| Age | 1.044 | (0.911, 1.196) | 1.168 | (0.934, 1.460) |
| Household Composition | 1.123 | (0.514, 2.451) | 0.582 | (0.184, 1.844) |
| Family in receipt of social welfare | 1.393 | (0.610, 3.183) | 1.073 | (0.320, 3.597) |
| Gender | 3.208* | (1.486, 6.928) | 0.972 | (0.398, 2.373) |
| MDM Decile | 1.021 | (0.898, 1.161) | 0.992 | (0.814, 1.210) |
| Natural Disaster | 0.363 | (0.005, 28.021) | 0.835 | (0.066, 10.573) |
| Stressful or scary medical procedure | 1.688 | (0.577, 4.937) | 1.778 | (0.424, 7.457) |
| War | n/a | n/a | 3.780 | (0.162, 88.108) |
| One Sexual Trauma | 4.108* | (1.466, 11.512) | 1.424 | (0.189, 10.722) |
| 2 or more sexual traumas | 15.595* | (4.050, 60.058) | 0.432 | (0.030, 6.124) |
| One violent trauma | 1.802 | (0.796, 4.081) | 1.528 | (0.484, 4.818) |
| Two or more violent trauma | 3.968* | (1.301, 12.096) | 5.062* | (1.113, 23.022) |
| One vicarious trauma | 0.624 | (0.280, 1.388) | 0.852 | (0.287, 2.532) |
| Two or more vicarious | 1.815 | (0.649, 5.076) | 0.211 | (0.008, 5.413) |

**Supplementary Table 7: One-Way Between Groups ANOVA Results of Differences in RCADS Subscale Scores Across Diagnostic Groups**

|  | No diagnosis (1) | CPTSD (2) | | | PTSD (3) | ANOVA | | | | | Post-hoc |
| --- | --- | --- | --- | --- | --- | --- | --- | --- | --- | --- | --- |
|  | Mean  (sd) | | Mean  (sd) | Mean  (sd) | | | F  (df) | p | ω2 | Tukey HSD | |
| Separation Anxiety Disorder (SAD) | 2.815  (3.252) | | 7.705  (4.796) | 4.421  (3.388) | | | 42.058  (2, 504) | <.001 | 0.14 | 2 > 3,1 | |
| Social Anxiety Disorder (SOC) | 10.853  (6.27) | | 19.818  (4.971) | 12.895  (5.353) | | | 43.068  (2, 504) | <.001 | 0.14 | 2 > 3,1 | |
| Obsessive Compulsive Disorder (OCD) | 4.840  (5.095) | | 15.318  (6.720) | 8.579  (6.569) | | | 62.287  (2,503) | <.001 | 0.20 | 2 > 3  2, 3 >1 | |
| Panic Disorder (PD) | 4.840  (5.095) | | 15.3182  (6.720) | 8.579  (6.569) | | | 80.418  (2, 503) | <.001 | 0.24 | 2 > 3  2, 3 >1 | |
| Generalized Anxiety Disorder (GAD) | 5.340  (3.882) | | 11.723  (3.991) | 8.316  (3.874) | | | 57.400  (2, 504) | <.001 | 0.24 | 2 > 3  2, 3 > 1 | |
| Major Depressive Disorder (MDD) | 7.323  (5.381) | | 18.023  (6.337) | 12.526  (5.461) | | | 81.993  (2, 503) | <.001 | 0.18 | 2 > 3  2, 3 > 1 | |
